# The rostromedial tegmental nucleus RMTg is not a critical site for ethanol-induced motor activation in rats

**DOI:** 10.1007/s00213-023-06425-4

**Published:** 2023-07-20

**Authors:** Claudia Esposito-Zapero, Sandra Fernández-Rodríguez, María José Sánchez-Catalán, Teodoro Zornoza, María José Cano-Cebrián, Luis Granero

**Affiliations:** 1 Departament de Farmàcia i Tecnologia Farmacèutica i Parasitologia, Facultat de Farmàcia, Universitat de València, Burjassot, Spain; 2 Lab of Functional Neuroanatomy (NeuroFun-UJI-UV), Unitat Predepartamental de Medicina, Faculty of Health Sciences, Universitat Jaume I, Castellón de la Plana, Spain

**Keywords:** Rostromedial tegmental nucleus, Locomotor activity, Salsolinol, Acetaldehyde, Brain metabolism of ethanol

## Abstract

**Rationale:**

Opioid drugs indirectly activate dopamine (DA) neurons in the ventral tegmental area (VTA) through a disinhibition mechanism mediated by mu opioid receptors (MORs) present both on the GABA projection neurons located in the medial tegmental nucleus/tail of the VTA (RMTg/tVTA) and on the VTA GABA interneurons. It is well demonstrated that ethanol, like opioid drugs, provokes VTA DA neuron disinhibition by interacting (through its secondary metabolite, salsolinol) with MORs present in VTA GABA interneurons, but it is not known whether ethanol could disinhibit VTA DA neurons through the MORs present in the RMTg/tVTA.

**Objectives:**

The objective of the present study was to determine whether ethanol, directly microinjected into the tVTA/RMTg, is also able to induce VTA DA neurons disinhibition.

**Methods:**

Disinhibition of VTA DA neurons was indirectly assessed through the analysis of the motor activity of rats. Cannulae were placed into the tVTA/RMTg to perform microinjections of DAMGO (0.13 nmol), ethanol (150 or 300 nmol) or acetaldehyde (250 nmol) in animals pre-treated with either aCSF or the irreversible antagonist of MORs, beta-funaltrexamine (beta-FNA; 2.5 nmol). After injections, spontaneous activity was monitored for 30 min.

**Results:**

Neither ethanol nor acetaldehyde directly administered into the RMTg/tVTA were able to increase the locomotor activity of rats at doses that, in previous studies performed in the posterior VTA, were effective in increasing motor activities. However, microinjections of 0.13 nmol of DAMGO into the tVTA/RMTg significantly increased the locomotor activity of rats. These activating effects were reduced by local pre-treatment of rats with beta-FNA (2.5 nmol).

**Conclusions:**

The tVTA/RMTg does not appear to be a key brain region for the disinhibiting action of ethanol on VTA DA neurons. The absence of dopamine in the tVTA/RMTg extracellular medium, the lack of local ethanol metabolism or both could explain the present results.

## Introduction

The rostromedial tegmental nucleus (RMTg), also known as the tail of the ventral tegmental area (tVTA), is a midbrain region posterior to the ventral tegmental area (VTA) that sends a dense GABAergic projection to midbrain dopamine (DA) neurons, including VTA and substantia nigra (Jhou et al. 2009; Kaufling et al. 2009). Due to the neuroanatomical position of the tVTA/RMTg and its relation with the lateral habenula and dopamine systems, this brain region has been mainly explored in the context of aversion and avoidance (Sánchez-Catalán et al. 2017; Jhou 2021; Vento et al. 2017; Proulx et al. 2018; Lammel et al. 2012; Stamatakis and Stuber 2012), anxiety and depression (Fu et al. 2019; Elmer et al. 2019; Li et al. 2022; Sun et al. 2020), and prediction error (Hong et al. 2011; Sánchez-Catalán and Barrot 2022). Moreover, it has been related with motor control (Bourdy et al. 2014; Faivre et al. 2020) and pain (Markovic et al. 2021). Finally, the tVTA/RMTg has been studied for its role in the affective state of drugs of abuse, such as opioids (Jhou et al. 2012; Kaufling and Aston-Jones 2015; Matsui and Williams 2011; Steidl et al. 2017; Wasserman et al. 2016), cocaine (Huff and LaLumiere 2015; Jhou et al. 2013; Parrilla-Carrero et al. 2021), and ethanol (Dornellas et al. 2021; Fu et al. 2016a, b; Glover et al. 2016; Sheth et al. 2016).

Recent studies demonstrate that tVTA/RMTg GABA neurons constitute a distinctive group of cells, different both in origin and functions from the VTA GABA interneurons (Lahti et al. 2016; Smith et al. 2019). Despite the differences between VTA GABA neurons and GABA neurons in the tVTA/RMTg, both subsets of neurons share two important characteristics: they show a high expression of mu opioid receptors (MORs) and exert a strong tonic and phasic inhibitory control on VTA DA firing (Jhou et al. 2012; Matsui et al. 2014; Matsui and Williams 2011; Lecca et al. 2011; Jalabert et al. 2011; Fields and Margolis 2015). Indeed, when directly applied in the tVTA/RMTg, opioid drugs, or more precisely, the agonists of MORs, can suppress the inhibitory control on VTA DA firing (Jalabert et al. 2011; Matsui and Williams 2011) in a manner equivalent, although more robust, to that reported by Johnson and North in their seminal paper (Johnson and North 1992). Matsui et al. (2014) compared the magnitude of the opioid-evoked inhibition of VTA DA neuron firing from each GABA afferent, showing that opioids reduce the inhibiting input from tVTA/RMTg more strongly than those from local VTA GABA interneurons. Thus, it has been suggested that the projection from the tVTA/RMTg could play a dominant role in acute opioid disinhibition of VTA DA neurons.

Like opioid drugs, ethanol seems to provoke its rewarding effects by interacting with MORs. To be more precise, it is not ethanol itself that interacts with the MORs but salsolinol, a secondary metabolite of ethanol, which behaves as an agonist of MORs (Xie et al. 2012). Racemic salsolinol can be locally formed in the brain after ethanol administration from the non-enzymatic condensation (Pictet–Spengler reaction) between acetaldehyde (ACH, the primary metabolite of ethanol) and DA (Hipólito et al. 2012), including the VTA (Bassareo et al. 2021). Behavioral and neurochemical data obtained in our laboratory and others, strongly suggest that direct administration of ethanol into the posterior VTA (pVTA) causes an increase in salsolinol levels (Bassareo et al. 2021) and, consequently, an intense activation of the VTA DA neurons (Sánchez-Catalán et al. 2009; Martí-Prats et al. 2010, 2013, 2015; Bassareo et al. 2021). In this sense, the pre-treatment with opioid-antagonists prevents the ability of ethanol to activate VTA DA neurons (Sánchez-Catalán et al. 2009), such as the blockade of the main metabolic systems for ethanol (i.e., catalase) (Marti-Prats et al. 2013; Bassareo et al. 2021). Moreover, direct administration of ACH into the pVTA is able to activate VTA DA neurons (Sánchez-Catalán et al. 2009) and sequestration of ethanol-derived ACH suppresses VTA DA neuron activation (Marti-Prats et al. 2010, 2013; Bassareo et al. 2021). On the other hand, the reduction of the synaptic availability in the VTA of DA impedes salsolinol formation after ethanol administration (Bassareo et al. 2021).

Hence, the ethanol-derived activation of VTA DA neurons seems to depend on (i) the existence of a local metabolism of ethanol that generates ACH and (ii) adequate levels of DA in the brain region considered. Therefore, for ethanol to be able to inhibit the GABAergic input that controls the activity of VTA DA neurons through the MORs, salsolinol must be formed previously and, for this, a double condition must be met: (i) There must be a local metabolism for ethanol that generates ACH and (ii) there must also be a sufficient concentration of DA to react with the ACH locally formed.

Considering the strong influence that the tVTA/RMTg exerts on VTA DA neurons and the high-level expression of MORs in the tVTA/RMTg GABA neurons, one important question arises at this point in the context of brain ethanol actions: could ethanol cause a disinhibition of VTA DA neurons if administered directly into the tVTA/RMTg? In other words, are the two necessary conditions mentioned above met in the tVTA/RMTg so that salsolinol can be formed and, therefore, the GABAergic projection from the tVTA/RMTg inhibited? If we assume that the GABAergic projection from the tVTA/RMTg, as indicated by Matsui’s data (Matsui et al. 2014), plays a crucial role in controlling the activity of VTA DA neurons, this question acquires a special importance. Thus, in the present study, we approach the analysis of this question. Our results suggest that (i) in the tVTA/RMTg the right conditions are not present for the formation of salsolinol after local ethanol administration and (ii) consequently, the tVTA/RMTg is not a critical site for ethanol-derived motor-activating effects in rats.

## Materials and methods

### Animals

Male Sprague-Dawley rats (280–300 g at the time of surgery) were used for our experiments. They were housed in plastic cages (42 × 27 × 18 cm^3^) in groups of four to six with controlled humidity and temperature (22°C), a 12:12-h light/dark cycle (on 08:00, off 20:00), and free access to food and water. All the procedures were carried out in strict accordance with the EEC Council Directive 86/609, Spanish laws (RD 1201/2005) and animal protection policies. Experiments were approved by the Animal Care Committee of the Faculty of Pharmacy at the University of Valencia, Spain (protocol codes: 2021/VSC/PEA/0078, approved on 30^th^ March 2021; 2022/VSC/PEA/0037/2, approved on 28^th^ March 2022).

### Drugs and chemicals

Ethanol was purchased from Scharlau (Madrid, Spain). ACH and [D-Ala2, N-Me-Phe4, Gly5-ol]-enkephalin (DAMGO; a selective agonist of the MORs) were purchased from Sigma Chemical Co. (St Louis, MO, USA). β-funaltrexamine (β-FNA; an irreversible antagonist of the MORs) was obtained from Tocris (Bristol, UK). Ethanol and ACH were freshly dissolved in artificial cerebrospinal fluid (aCSF)/ascorbate solution prior to use. The aCSF/ascorbate solution consisted of 120.0 mM NaCl, 4.8 mM KCl, 1.2 mM KH2PO4, 1.2 mM MgSO4, 25.0 mM NaHCO3, 1.2 mM CaCl2, 100 mM D-glucose, and 0.2 mM ascorbate and pH was adjusted to 6.5. Stock solutions of DAMGO and β-FNA were prepared by dissolving the compound in the proper volume of distilled water. These solutions were then kept frozen at −40°C as aliquots until use. Prior to use, aliquots of the stock solutions were conveniently diluted with aCSF/ ascorbate solution to obtain the appropriate concentration. The final pH for the ACH solutions was between 6.4 and 6.6. All the other reagents used were of the highest commercially available grade.

### Surgery and post-surgical care

All surgeries were performed under isoflurane anesthesia (1.5–2 minimum alveolar concentration, MAC) and under aseptic conditions. Rats received 2.5 mg/kg of carprofen (s.c) and 0.1% topical lidocaine in the surgical area and in the ears before surgery. Then, animals were placed in a stereotaxic apparatus (Stoelting, USA) and an incision (8–10 mm) was made in the skin above the skull. Three holes were drilled: two for the skull screws and the other for the guide cannulae (Plastics One, USA). Each animal was implanted unilaterally with one 28-gauge guide cannula aimed at 1.0 mm above the tVTA/RMTg. The coordinates relative to bregma and skull surface (Paxinos and Watson 2007) were as follows: A/P −6.9 mm; L ± 1.4 mm; D/V −7.4 mm. Cannulae were angled toward the midline at +6° from the vertical. Cannulae assemblies were secured in place with dental cement. A stainless steel stylet (33-gauge), extending 1.0 mm beyond the tip of the guide cannula, was put in place at the time of surgery and removed at the time of testing. After surgery, rats were housed in individual rectangular plastic cages (42 × 27 × 18 cm^3^, located side by side in order to prevent the influence of chronic stress on performance due to isolation) with free access to food and water for 7–10 days.

### Drug microinjection procedures

All the intra-tVTA/RMTg drug microinjections were carried out with 33-gauge stainless steel injectors, extending 1.0 mm below the tip of the guide cannula. Injectors were attached to a 25 μL Hamilton syringe by using PE-10 tubing. Microinjections were carried out using a syringe pump (Kd Scientific) which was programmed to deliver a total volume of 300 nL (0.15 μl/min) when the pre-treatment (aCSF or β-FNA) was administered, or 200 nL (0.6 μl/min) in the case of the treatment (aCSF, EtOH, ACH or DAMGO). This procedure of administration of the pharmacological agents was identical to that previously used in our previous experiments in the pVTA (Martí-Prats et al. 2010; Sánchez-Catalán et al. 2009). Following the infusion, the injector remained in place for 1 min to allow the diffusion of the drugs, and it was then removed, the stylet was replaced, and the locomotor activity was registered when appropriate. All the injections were carried out in the experimental room.

### Handling and test procedure

After 24 h of post-surgical recovery, animals were taken from the colony, brought to the experimental room, and handled for 10 min/day until the experimental day. During this phase, animals became accustomed to the experimenter, the experimental room, and to the injection procedure with a total of four to seven sessions to decrease the activation effects of the manipulations taking place during the injection process, as well as the novelty-activating effects of the testing room. Tests were performed 7–10 days after surgery. On the last habituation day, each animal was also placed in its experimental cage (42 × 27 × 18 cm^3^) for 30 min to decrease the novelty-activating effects of the testing cage. On the day of the experiment, rats were again taken from the colony room and brought to the experimental room 20–30 min prior to the start of the session, in the same rectangular cages in which the animals were housed. After this initial period, experiments started according to the protocol described in the drug microinjection procedures. Animals were placed in the experimental cage immediately after injection (maximum latency time of 10 s). All the experiments were recorded by a digital video camera for 30 min, and the distance traveled (centimeters) during those 30 min was analyzed for that 30 min using the Raddot program (Universitat de València, Spain). For the handling and test, the experimental room was illuminated with soft white light.

### Experiments

Three experiments were conducted:

### Experiment 1. Intra-tVTA/RMTg injections of DAMGO and β-FNA+DAMGO

This first experiment had the purpose of verifying, under our experimental conditions, that the local administration of DAMGO into the tVTA/RMTg could cause a MORs-dependent motor activation in the treated animals. We used the same doses of DAMGO and β-FNA that we had previously used in the pVTA (Sánchez-Catalán et al. 2009). We could observe in that paper, that 0.13 nmol DAMGO triggered an intense activation after intra-VTA administration. In the same manner, the β-FNA dose (2.5 nmol) was able to significantly reduce the activation induced by 0.13 nmol DAMGO.

Twenty-eight rats distributed into four subgroups of rats (*n*=7) were used for the present experiment. The day before the experiment, animals received a pre-treatment with either aCSF or β-FNA (2.5 nmol) depending on the experimental subgroup. On the day of the experiment, rats received a unilateral microinjection of aCSF or DAMGO (0.13 nmol). Thus, the four subgroups formed were: aCSF+aCSF; aCSF+DAMGO; β-FNA+aCSF; β-FNA+DAMGO.

### Experiment 2. Intra-tVTA/RMTg injections of ethanol

In our previous papers (Marti-Prats et al. 2010; Sánchez-Catalán et al. 2009), we showed that the intra-pVTA administration of ethanol, at doses of 75 nmol or 150 nmol, increased the motor activity of Wistar rats. In those experiments, injections of ethanol into the pVTA increased the locomotor activity of rats with maximal effects at doses of 150 nmol. These motor-activating effects were significantly reduced by previously administering β-FNA (2.5 nmol) into the pVTA which suggested the involvement of MORs in the motor-activating responses evoked by ethanol. In the present study, we decided to reproduce these results in the tVTA/RMTg using experimental conditions practically identical to those previously used in the pVTA. Therefore, we decided to test the ability of 150 nmol and 300 nmol ethanol directly administered into the tVTA/RMTg to evoke a MORs-dependent motor activation in rats.

Twenty-one Sprague-Dawley rats were randomly assigned to one of the three experimental subgroups (*n*=7/subgroup). Each animal received two intra-tVTA/RMTg microinjections on consecutive days. On day 1, animals received aCSF, ethanol 150nmol or ethanol 300 nmol depending on the experimental subgroup and motor activity was recorded. On day 2, all animals, regardless of the subgroup to which they belonged, received an intra-tVTA/RMTg microinjection of 0.13 nmol of DAMGO and the locomotor activity of the animals was evaluated once more.

### Experiment 3. Intra-tVTA/RMTg injections of ACH

In our previous paper (Sánchez-Catalán et al. 2009), we showed that the intra-pVTA administration of ACH, at doses 250 nmol, increased the motor activity of Wistar rats. In the present study, we decided to test the locomotor-activating effects of this ACH dose after intra-tVTA/RMTg administration. Sixteen Sprague-Dawley rats were randomly assigned to one of the two experimental subgroups (*n*=7). Each animal received two intra-tVTA/RMTg microinjections on consecutive days. On day 1, animals received aCSF or ACH 250 nmol, depending on the experimental subgroup and motor activity was recorded as indicated above. On day 2, all animals, regardless of the subgroup to which they belonged, received an intra-tVTA/RMTg microinjection of 0.13 nmol of DAMGO and the locomotor activity of the animals was assessed.

### Histological validation of the cannula placements

**Fig. 1:**
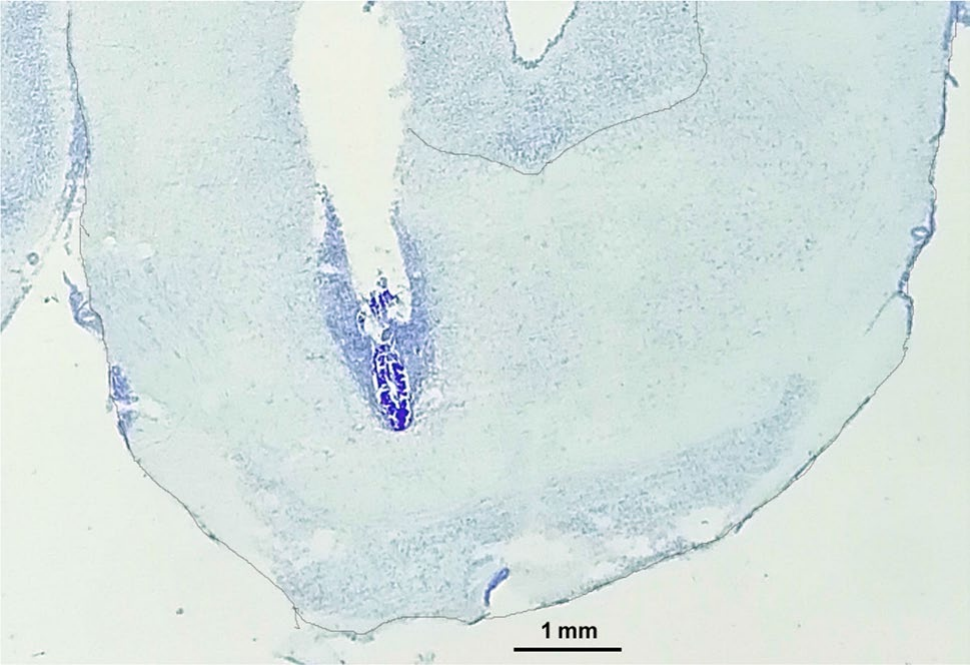
Representative photomicrograph showing a 40-μm-thick coronal brain section stained according to the cresyl violet procedure from a rat belonging to the aCSF+ethanol (150 nmol) group in experiment 2. Scale bar=1 mm

At the end of the experiments, the rats were sacrificed by pentobarbital overdose (90 mg/kg) and the brain was freshly removed and quickly frozen using dry ice. The 40-μm-thick coronal sections of the brain were obtained using a cryostat. Sections were used for the verification of the cannula placements of all the datasets presented in this study. To that end, coronal sections of the tVTA/RMTg were mounted and stained according to the cresyl violet protocol. Then, the location of the cannula tips was carefully examined by a researcher, who was unaware of the experimental condition of the animals, using optical microscopy. A representative photomicrograph is shown in Fig. 1.

### Statistical methods

A two-way ANOVA (2 × 2) with interaction was used to analyze the distance traveled by animals across the 30-min sessions in experiment 1. Factors analyzed were “pre-treatment” (aCSF or β-FNA) and “treatment” (aCSF or DAMGO). Post hoc comparisons were made using an adjusted Bonferroni’s test. A mixed two-way ANOVA was used to compare the motor activities obtained in experiments 2 and 3. In both cases, between factor was “treatment on day 1” (with three levels in experiment 2: aCSF, ethanol 150 nmol and ethanol 300 nmol, or two levels in experiment 3: aCSF and ACH 250 nmol) and the within factor was “day of treatment” (with two levels for both experiments: day 1 and day 2). The level of significance was always set at *p*<0.05. All the analyses were done using SPSS, v. 15.0.

## Results

### Experiment 1. Intra-tVTA/RMTg injections of DAMGO and β-FNA+DAMGO

Our aim in the present experiment was to confirm that, under our experimental conditions, 0.13 nmol DAMGO can produce a significant motor activation in rats after local intra-tVTA/RMTg administration. It is important to remark that it was previously demonstrated that this dose of DAMGO was able to produce a robust motor activation after intra-pVTA administration (Sánchez-Catalán et al. 2009; Marti- Prats et al. 2010).

**Fig. 2:**
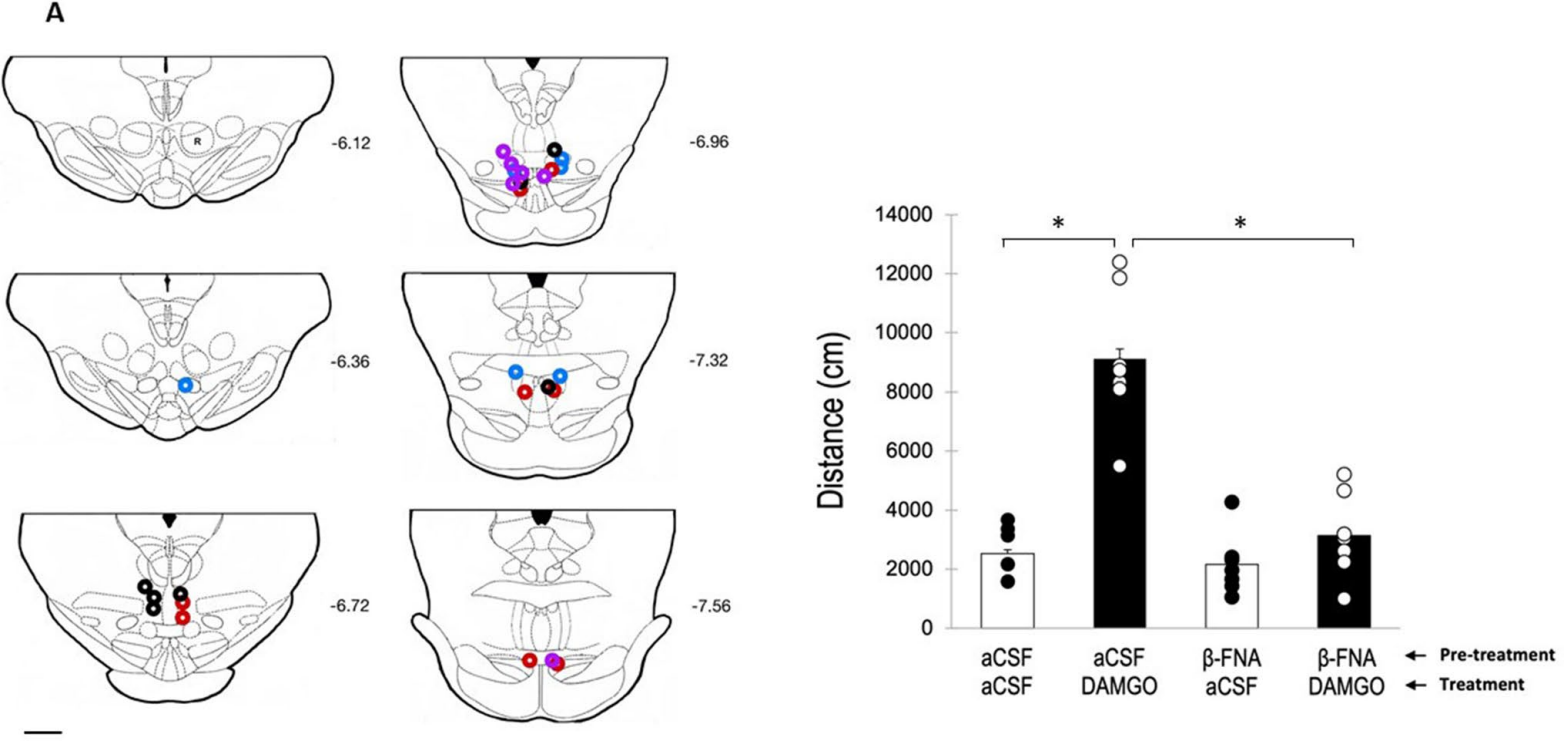
Effect of intra-tVTA/RMTg administration of DAMGO on motor activity of rats. Panel **A**: diagram of coronal sections of the brains of the rats used in experiment 1 showing the placement of the tip of the injection cannulae (aCSF+aCSF (blue), aCSF+DAMGO (red), β-FNA+aCSF (black), β-FNA+DAMGO (violet)). Numbers indicate distance in mm from Bregma (adapted from Paxinos and Watson 2007). Scale bar=1 mm. Panel **B**: distance traveled (mean±SEM) in 30 min by rats pre-treated with aCSF or β-FNA (2.5 nmol) 1 day before DAMGO (0.13 nmol) or aCSF treatment. A single asterisk (*) indicates significant differences (*p*<0.05)

Cannula placements in animals used in this experiment are shown in Fig. 2. As can be seen, all animals showed correct placements according to the anatomic characterization of the rat tVTA/RMTg by Smith et al. (2019). Distances traveled by animals used in this experiment are also shown in Fig. 2. As can be observed, only animals pre-treated with aCSF and treated with 0.13 nmol of DAMGO directly into the tVTA/RMTg showed a significant locomotor activation. Two-way ANOVA confirmed these observations. Both the main effects for pre-treatment (*F*(1,24)=29.975; *p*<0.001) and treatment (*F*(1,24)=42.623; *p*<0.001) were statistically significant. In the same manner, the interaction pre-treatment × treatment was also statistically significant (*F*(1,24)=23.361; *p*<0.001). Post hoc comparisons confirmed that the mean distance traveled by animals treated with aCSF/DAMGO differ from acSF/aCSF (*p*<0.001). Moreover, aCSF/aCSF did not differ between the β*-FNA*/aCSF group (*p*=0.654), suggesting that pre-treatment with β*-FNA* did not modify basal activity of the animals. Moreover, pre-treatment with β*-FNA* prevented the increase in motor activities of the animals treated with DAMGO (aCSF/DAMGO vs. β*-FNA*/DAMGO) (*p*=0.242). Thus, these results confirm that DAMGO, directly administered into the tVTA/RMTg, is able to increase the motor activity of rats, through its interaction with MORs.

### Experiment 2. Intra-tVTA/RMTg injections of ethanol

To test if the tVTA/RMTg is, as occurs with the pVTA, a critical region mediating the ethanol activating effects of the VTA DA neurons, we proceeded to administer on day 1 of the experiment, either 150 or 300 nmol of ethanol directly into the tVTA/RMTg. All animals showed correct cannulae placements after histological examination according to the anatomic characterization of the rat tVTA/RMTg by Smith et al. (2019). In order to have pharmacological (in addition to histological) confirmation of the position of the injection cannulae, all the animals used in this experiment received on day 2, a dose of 0.13 nmol of DAMGO which, as we demonstrated in experiment 1, is able to significantly increase the motor activity of the animals.

**Fig. 3:**
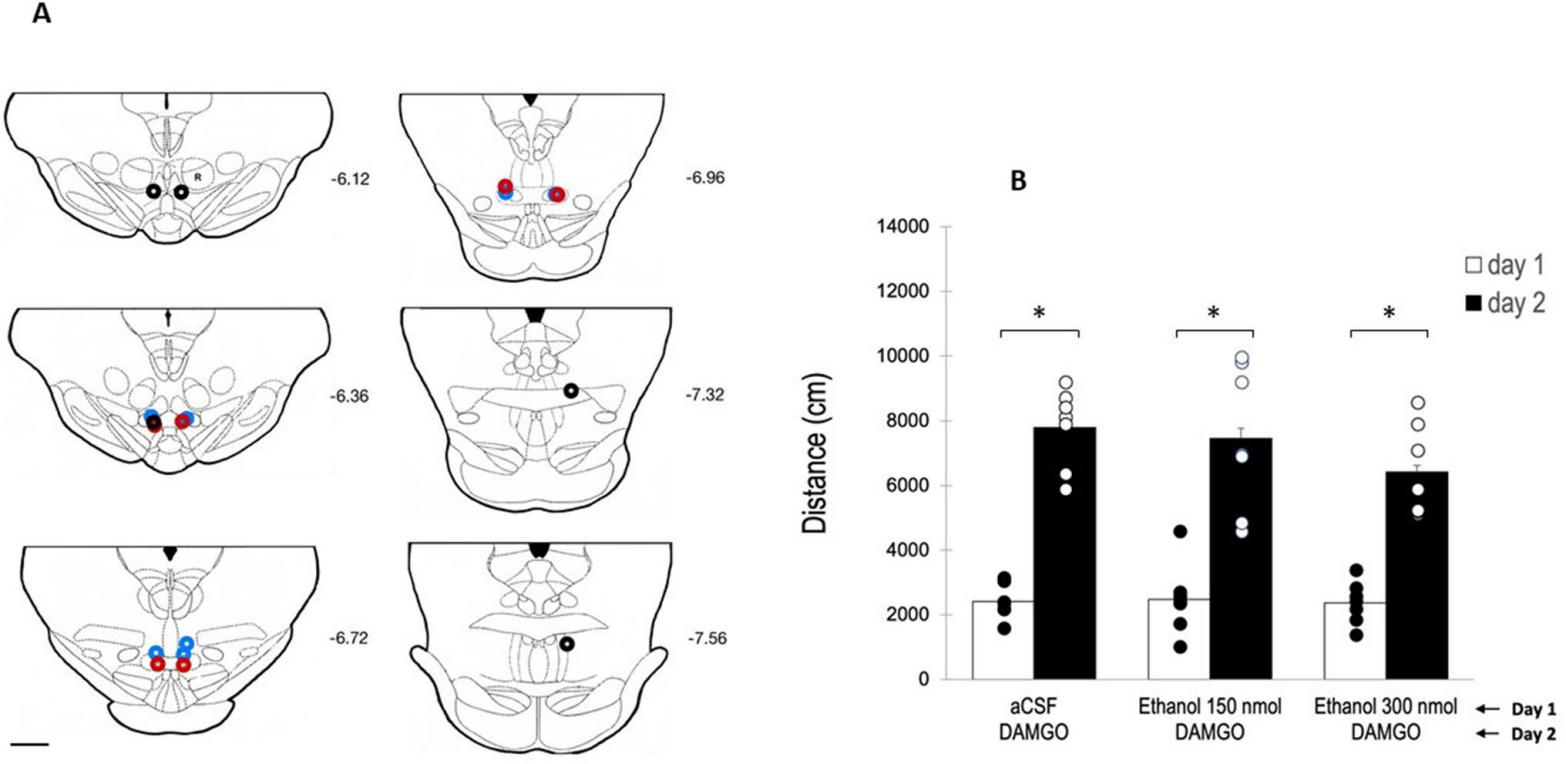
Effect of intra-tVTA/RMTg administration of ethanol on motor activity of rats. Panel **A**: diagram of coronal sections of the brains of the rats used in experiment 2 indicating the placement of the tip of the injection cannulae (aCSF+aCSF (blue), aCSF+ethanol (150 nmol) (red), aCSF+ethanol (300 nmol) (black)). Numbers indicate distance in mm from Bregma (adapted from Paxinos and Watson 2007). Scale bar=1 mm. Panel **B**: distance traveled (mean±SEM) in 30 min on day 1 by rats treated with aCSF, ethanol 150 nmol or ethanol 300 nmol (white bars); and on day 2 by rats treated with DAMGO (0.13 nmol) (black bars). A single asterisk (*) indicates significant differences (*p*<0.05)

Results of this experiment are shown in Fig. 3. As can be observed, neither the 150 nmol dose nor the 300 nmol dose of ethanol significantly altered the motor activity of rats on day 1 of the experiment, as compared with activity obtained in the aCSF treated subgroup of animals. The mixed two-way ANOVA with interaction indicated that neither the main effects for the between factor (*F*(2,18)=1192; *p*=0.327) nor the interaction ((*F*(2,18)=1192; *p*=0.327) were statistically significant. However, the main effects for the within factor were statistically significant (*F*(1,18)=179,181; *p*<0.001), being higher on day 2 (DAMGO) than on day 1.

Therefore, our results show that ethanol directly administered into the tVTA/RMTg was not able to significantly increase motor activity of the animals. It is unlikely that this finding is due to improper positioning of the injection cannulae since both the histological analysis of the sections (Fig. 3A) and the administration of DAMGO on day 2 of the experiment (administration that led, as we have seen, to highly significant increases in the distance traveled by the animals) suggest that the tips of the cannulae were located in the tVTA/RMTg.

### Experiment 3. Intra-tVTA/RMTg injections of ACH

In this experiment, we proceeded to administer on day 1 of the experiment, 250 nmol of ACH directly into the tVTA/RMTg. Previous experiments (Sánchez-Catalán et al. 2009) demonstrated that this dose of ACH is able to produce a significant activation of rats when administered into the pVTA. Histological examination of the brains from animals used in this experiment revealed that all animals showed correct placements according to the anatomic characterization of the rat tVTA/RMTg by Smith et al. (2019). As in experiment 2, in order to have an additional pharmacological confirmation of the position of the injection cannulae, all the animals used in this experiment received on day 2, a dose of 0.13 nmol of DAMGO which, as we demonstrated in experiment 1, is able to significantly increase the motor activity of the animals.

**Fig. 4:**
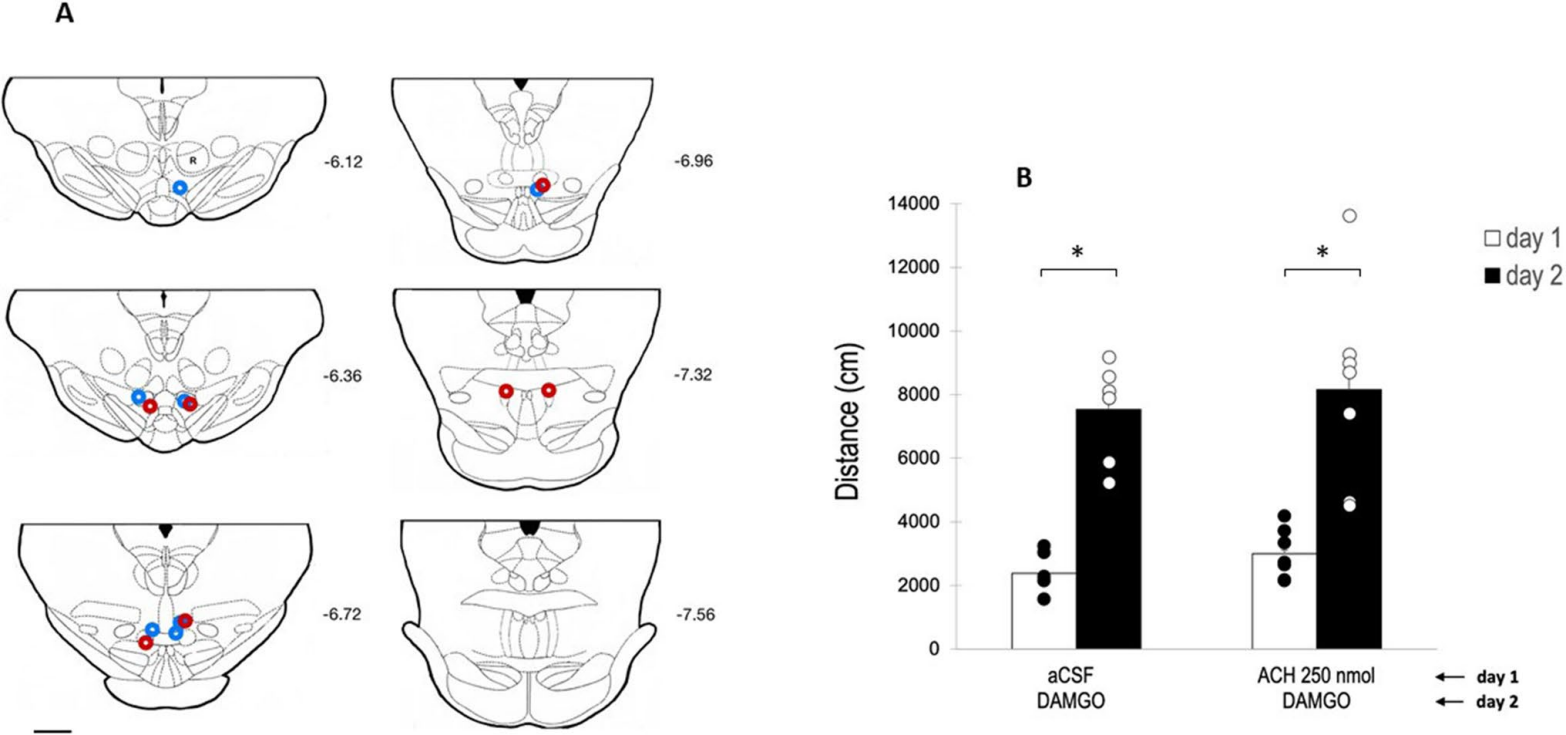
Effect of intra-tVTA/RMTg administration of ACH on motor activity of rats. Panel A: diagram of coronal sections of the brains of the rats used in experiment 3 indicating the placement of the tip of the injection cannula (aCSF+aCSF (blue), aCSF+ACH (250 nmol) (red)). Numbers indicate distance in mm from Bregma (adapted from Paxinos and Watson 2007). Scale bar=1 mm. Panel **B**: white bars: distance traveled (mean±SEM) in 30 min on day 1 by rats treated with aCSF or ACH 250 nmol (white bars); and on day 2 by rats treated with DAMGO (0.13 nmol) (black bars). A single asterisk (*) indicates significant differences (*p*<0.05)

Results of this experiment are shown in Fig. 4. As can be observed, ACH 250 nmol did not significantly alter the motor activity of rats on day 1 of the experiment, as compared with motor activity measured in the aCSF-treated subgroup of animals. The mixed two-way ANOVA with interaction indicated that neither the main effects for the between factor (*F*(1,12)=0.072; *p*=0.792) nor the interaction ((*F*(1,12)=0.070; *p*=0.797) were statistically significant. However, the main effects for the within factor (“day of treatment”) were statistically significant (*F*(1,12)=56.421; *p*<0.001), being higher on day 2 (DAMGO) than on day 1.

Our results show that ACH directly administered into the tVTA/RMTg was not able to significantly increase motor activity of the animals. As indicated above for experiment 2, it is unlikely that this finding is due to improper positioning of the injection cannulae since both the histological analysis of the sections (Fig. 4A) and the administration of DAMGO on day 2 of the experiment suggested that the tips of the cannulae were correctly located in the tVTA/RMTg.

## Discussion

The results of the present study suggest that the tVTA/RMTg is not a relevant brain region to explain the motor-activating effects of ethanol in the midbrain region through a disinhibition of the VTA DA neurons. As our results show, neither ethanol nor its main metabolite, ACH, locally administered into the tVTA/RMTg at doses that were clearly motor-activating in pVTA (Sánchez-Catalán et al. 2009), were capable of increasing the exploratory motor activity of the rats.

Several research groups have evidenced in the last decade the importance of the tVTA/RMTg and their GABA neurons in controlling the midbrain DA neuron activity (Sanchez-Catalan et al. 2014). At present, it is clear that the activation or inhibition of the tVTA/RMTg potently inhibits or activates, respectively, the activity of midbrain DA neurons (Hong et al. 2011; Kaufling and Aston-Jones 2015; Jalabert et al. 2011; Bourdy et al. 2014; Lecca et al. 2011). As described in the introduction section, the tVTA/RMTg participates in the affective states induced by some drugs of abuse. Specifically, the agonists of MORs mediate an inhibition of the tVTA/RMTg GABA neurons, disinhibiting VTA DA neurons (Jhou et al. 2012; Matsui et al. 2014; Matsui and Williams 2011; Lecca et al. 2011; Jalabert et al. 2011). This indirect activation of the DA system has important functional consequences; for example, this disinhibition helps to explain why rats self-administer MOR agonists into the tVTA/RMTg (Jhou et al. 2012). All the above data demonstrating the existence of this inhibitory control of the tVTA/RMTg over VTA DA neurons has served to review the circuits and the mechanisms involved in the acute and chronic actions of opiate drugs of abuse on the mesolimbic DA neurons (Barrot et al. 2012; Bourdy and Barrot 2012; Kaufling and Aston-Jones 2015). Likewise, the indirect activation of the DA systems also explains why temporary inactivation or injury of the tVTA/RMTg increases exploratory locomotor activity (Lavezzi et al. 2015; Vento et al. 2017). It is noteworthy that the activation of the tVTA/RMTg decreases the consumption and preference for ethanol (Fu et al. 2016a), whereas lesions or pharmacological inhibition (muscimol) of the tVTA/RMTg increase the intake and preference for ethanol (Fu et al. 2016a, b; Sheth et al. 2016).

On the other hand, ethanol is another drug of abuse that seems to exert its acute activating actions on mesolimbic DA neurons through MORs. It has been suggested that actions of ethanol on pVTA DA neurons depend on the interaction of salsolinol (a secondary metabolite of ethanol) with MORs located in the pVTA GABA interneurons (Sánchez-Catalán et al. 2009; Xie et al. 2012; Hipólito et al. 2012; Martí-Prats et al. 2013, 2015; Bassareo et al. 2021). Thus, it seemed of great interest to us to explore whether acute local administration of ethanol into the tVTA/RMTg could, as occurs with classical opiate agonists, trigger a behavioral activation in rats.

Our results show that, contrarily to what has been described for opiates, ethanol directly administered into the tVTA/RMTg was unable to evoke motor activations in the experimental animals. Since, for ethanol to be capable of activating MORs, it must first be biotransformed into salsolinol, it seems logical to explore whether this lack of efficacy to induce motor activation in rats could be due to the lack of salsolinol formation in the tVTA/RMTg after local ethanol administration. The absence of local ethanol metabolism could be one of the explanations for the lack of salsolinol formation: if the enzyme systems necessary to generate ACH do not exist in the tVTA/RMTg, salsolinol cannot be produced. However, there is another possibility that could also help to explain why salsolinol could not be generated after local administration of ethanol: the lack of adequate levels of DA in the tVTA/RMTg. Salsolinol is formed by a non-enzymatic Pictet–Spengler reaction between ACH and DA (Hipolito el al. 2012). Consequently, if DA levels in the tVTA/RMTg are too low or non-existent, the local formation of salsolinol cannot occur, even though the enzyme systems to oxidize ethanol to ACH were present in the tVTA/RMTg.

To explore this intriguing possibility, we decided to administer 250 nmol of ACH directly into the tVTA/RMTg in experiment 3. The rationale of this experiment is obvious: if the lack of effects detected after ethanol administration in experiment 2 was due to the lack of local ethanol metabolism, the direct administration of ACH should be able to overcome this deficiency and cause an increase in the motor activity of the animals as long as tVTA/RMTg DA extracellular levels were adequate to enable salsolinol formation. It is important to remember that, as we demonstrated in previous papers (Sánchez-Catalán et al. 2009), when ACH is directly administered into the pVTA (a brain region with high DA extracellular levels (Adell and Artigas 2004)), animals display a significant motor activation, similar to that observed after ethanol microinjection. Results from our present experiment, however, clearly show that administration of ACH into the tVTA/RMTg does not produce, contrary to what has been observed in the pVTA, any significant change in the motor activity of rats. Obviously, these results, per se, do not support or rule out the existence of ethanol metabolism in the tVTA/RMTg, but they strongly suggest that the lack of motor-activating effects after intra-tVTA/RMTg ethanol administration could be due to the absence of adequate levels of DA in the tVTA/RMTg to generate salsolinol.

## Conclusions

It has been suggested that the tVTA/RMTg may be crucial in the context of the mechanisms through which different drugs of abuse, particularly opiates, activate the VTA DA neurons (Barrot et al. 2012; Jhou 2021). Opiates do not need to be biotransformed to interact with their respective receptors, which are abundantly expressed in tVTA/RMTg GABA neurons, making this brain region a key for understanding the mechanism used by these drugs of abuse to activate (disinhibit) midbrain DA neurons. However, ethanol requires both a previous oxidation and condensation of its first metabolite (ACH) with DA to generate the salsolinol molecule, which is ultimately responsible for the interaction with the MORs. According to our results, tVTA/RMTg does not seem to meet the appropriate neurochemical conditions for this to occur.
